# Evaluating the knowledge on microbiome and dysbiosis in allergic diseases among medical sciences students in Saudi Arabia

**DOI:** 10.1186/s12948-022-00168-x

**Published:** 2022-01-31

**Authors:** Aisha Alamri, Suzan A. AlKhater

**Affiliations:** 1 Department of Clinical Laboratory Sciences, College of Applied Medical Sciences, Imam Abdulrahman Bin Faisal University, Dammam, Saudi Arabia; 2 College of Medicine, Imam Abdulrahman Bin Faisal University, Dammam, Saudi Arabia; 3 Department of Pediatrics, King Fahad Hospital of the University, Al-Khobar, Saudi Arabia

**Keywords:** Dysbiosis, Human microbiome, Knowledge, Microbiology, Medical students

## Abstract

**Background:**

Microbiome science deals with the development of diseases that are derived from the interaction between the host immune system and microbes. Microbiome disturbance or dysbiosis has been increasingly recognized as an important contributor to the pathogenesis of allergic diseases. Thus, this field is pivotal in the management of allergic disorders. Despite the increasing prevalence of allergic disorders in Saudi Arabia, medical students lack knowledge of microbiome science. Therefore, this study aimed to assess the level of knowledge of medical sciences students on the human microbiome, dysbiosis, and management of the impaired microbiome with a focus on allergic diseases and asthma.

**Methods:**

An online survey was designed, validated, and distributed to 100 final-year students and interns majoring in clinical nutrition, public health, and clinical laboratory sciences at a single university in Saudi Arabia. The study period was from November 2020 to January 2021.

**Results:**

The overall knowledge of the human microbiome was adequate among the participants, but their understanding of dysbiosis and management of the impaired microbiome was low to moderate. Knowledge of dysbiosis management was significantly higher in students majoring in clinical nutrition than in those majoring in public health and clinical laboratory sciences.

**Conclusions:**

Collectively, this study provides the first evidence that knowledge of specific domains of microbiome science among a cohort of medical sciences students in Saudi Arabia is insufficient. Large-scale studies are warranted to confirm these observations at a national level, and specific curriculum modifications are necessary to improve the knowledge of future healthcare professionals about clinical applications of microbiome science.

**Supplementary Information:**

The online version contains supplementary material available at 10.1186/s12948-022-00168-x.

## Background

Microbes are unicellular and multicellular organisms which could only be visualized under a microscope and may include bacteria, fungi, archea, and protists [1]. Various microbial species colonize the internal and external surfaces of the human body [2]. Pioneering studies have led to the foundation of a field called human microbiome science, which focuses on the functional investigation of microbial communities in different mucosal surfaces (such as the skin and respiratory and intestinal tracts) during homeostasis as well as non-infectious disease development [3]. For instance, following the establishment of the Human Microbiome Project in 2008 and various technological advances in life science research [4, 5], multiple lines of evidence have suggested that microbial communities that populate the body during the early years of life are vital for the development of the immune system and the regulation of inflammatory responses [3]. Additionally, microbiome disturbance or dysbiosis, which often results from the misuse of antibiotics and specific pathological conditions, has been increasingly recognized as an important contributor to disease pathogenesis [6, 7]. In this regard, many studies have investigated the role of microbes in allergic disorders [8, 9], metabolic syndromes [10, 11], and neurological illnesses [12, 13]. Conversely, the potential therapeutic use of beneficial bacterial strains in the form of nutritional supplements (probiotics) or via a more complex intervention, such as fecal microbiota transplantation (FMT), to restore microbial balance, thereby managing disease conditions, has been explored in several inflammatory and metabolic disorders with promising results [14–17]. Collectively, this evidence from basic science and clinical studies not only highlights a previously underappreciated role of the microbiome in health and diseases but also emphasizes an important emerging need to provide healthcare professionals with updated medical education on these topics.

Few studies have examined the knowledge of the public and healthcare professionals worldwide on human microbiome science. A study conducted in the United Arab Emirates (UAE) reported that knowledge of the microbiome is generally low and positively correlated with the status and level of education of health professionals [18]. More importantly, the study found that antibiotics were improperly administered, which could have resulted in dysbiosis. Another study in the United States (US) surveyed dental students and revealed that while most of them were confident about their oral microbiome knowledge, their average score for the study was only 35.2% [19]. In contrast, students in the Philippines were found to have adequate knowledge of probiotics, but this was not associated with their consumption of probiotic foods [20]. Similarly, medical sciences students in Iran also had adequate general knowledge about probiotics, but their awareness of commercially available products and, consequently, their consumption of these items was low [21]. Concerning FMT, a survey of medical students in Romania revealed that only one-third of the participants had an acceptable level of knowledge about this procedure [22]. FMT knowledge in a large cohort of Chinese medical students also appeared to be insufficient [23]. These studies suggest that while medical sciences students have some understanding of microbiome science, their knowledge about specific domains of this field, particularly those with clinical importance such as the usage of antibiotics, probiotics, and FMT, is inadequate.

In Saudi Arabia, the prevalence of several health conditions, such as metabolic syndromes and allergic diseases, where the microbiome plays a pivotal role in disease initiation and progression, has rapidly increased in recent years [24–28]. However, to date, no studies have assessed the knowledge of microbiome science in healthcare professionals and students whose current and prospective jobs include managing medical and nutritional health conditions at the community level and raising public awareness of diseases of important public health concern such as allergic disorders and metabolic syndromes. Notably, while medical students in Saudi Arabia take microbiology courses as part of their training, the content of these courses primarily focuses on host–pathogen interaction from an infectious disease perspective. Many recent terminologies and concepts of microbiome science, such as dysbiosis and FMT, and the concept of the microbiome as a therapeutic or disease-promoting agent in these non-infectious contexts are not formally introduced in most of these courses. Consequently, the students are not expected to be well informed about the latest advances in microbiome science and its clinical application. Therefore, the current study aimed to evaluate the knowledge of medical sciences students in Saudi Arabia on microbiome science with a focus on allergic diseases and asthma.

The study was conducted in a cohort of medical sciences students in three major subjects at the Imam Abdulrahman Bin Faisal University: clinical laboratory sciences (CLS), clinical nutrition (CN), and public health (PH). These majors were selected for their scientific and clinical relevance to the microbiome. For CLS students, this knowledge provides critical insights for evaluating commensals vs. pathogenic microbes in clinical isolates and their relevance to patients’ health status. For CN and PH students, this body of information is essential for developing nutritional programs and public health policies, respectively, to improve health and prevent disease. The study outcome is anticipated to provide important insights for medical curriculum development with a focus on the clinical application of microbiome sciences to address emerging public health concerns in Saudi Arabia.

## Methods

### Study design

This cross-sectional observational study aimed to evaluate knowledge and perception about the microbiome and dysbiosis in health and disease among final-year students and interns (recent graduates who engage in 1 year of supervised practical training in a laboratory or hospital setting) in medical sciences majors at various colleges of the Imam Abdulrahman bin Faisal University, Dammam City, Saudi Arabia, from November 2020 to January 2021. All selected students signed informed consent forms before participating in the survey. Participants who consented were provided with instructions to complete an online questionnaire via a shared QuestionPro^®^ link. The study was approved by the Institutional Review Board of Imam Abdulrahman Bin Faisal University (IRB-2021-03-072).

### Inclusion criteria

A total of 100 female students majoring in CLS and CN at the College of Applied Medical Sciences and PH at the College of Public Health were recruited for the study with the assistance/supervision of the academic advisor and/or the class leader of each major. The participants had varying degrees of exposure to topics related to the microbiome in different microbiology courses taught by instructors with a minimum of a Ph.D. degree in medical microbiology and clinical laboratory sciences. CLS students completed four courses: Introduction to Clinical Microbiology, Clinical Microbiology II, Diagnostic Microbiology, and Parasitology and Virology. CN students were trained in microbiology and functional food, while PH students were trained in microbiology as well as parasitology and virology). The microbiome was covered in approximately 15–20% of the said courses for CLS students and 5–10% for CN and PH students.

### Survey development and validation

The survey was developed based on the expertise in microbiome science and knowledge of the clinical management of the microbiome of the authors, as well as through a literature review of important topics related to the microbiome. Subsequently, it was validated by three microbiology course experts, and no major modifications were required. The structured questionnaire consisted of three sections, assessing students’/interns’ knowledge of (1) microbiome science, which focuses on the concept of the microbiome in different parts of the body, (2) impaired microbiota and dysbiosis, which focuses on changes in the microbiome in relation to disease conditions, and (3) the management of dysbiosis, which focuses on two therapeutic approaches to manipulate the microbiome (probiotic usage and FMT) (Additional file 1).

### Data collection and statistical analysis

The data were collected online using a QuestionPro^®^ link, and correct answer rates for each question and topic were calculated. The knowledge levels were defined as adequate (75–100% students per group with correct responses), moderate (50–74% students per group with correct responses), and low (< 50% students per group with correct responses) as previously described [29]. The data were analyzed using IBM SPSS Statistics v26 2019. The differences in knowledge levels among the three student groups were analyzed using chi-square tests. P values  < 0.05 were considered statistically significant.

## Results

A total of 100 female participants, aged 21–23 years including 32 CN students, 37 CLS students, and 31 PH students, responded to the survey (75% response rate). Additionally, 53% of respondents were final-year students, and 47% were interns. Since both final-year students and interns with the same majors had similar exposure to the microbiology courses, these two groups of students were considered one sample representing the relevant major for further analyses.

### Knowledge of microbiome science

Overall knowledge of microbiome science was adequate among the three medical sciences majors, with CLS having the highest percentage of students with adequate knowledge (83.8%). However, no significant differences in overall knowledge level were found among CN, CLS, and PH students (Table 1). Concerning the specific concepts in the domain of microbiome science, 73% of all students provided correct answers to the questions related to the microbiome concept (Table 2). However, only 49% of the participants correctly answered that the term “microbiome” refers to the total collection of microorganisms in the body. Consistent with this finding, 42% of participants correctly answered that this term does not refer to specific bacterial species populating various body compartments. Notably, while most of the participants correctly agreed that the gastrointestinal tract, skin, and vagina are covered by microorganisms (98%, 95%, and 88%, respectively), only two-thirds (66%) of participants correctly indicated that microorganisms could be found in the respiratory tracts (Table 2).

**Table 1:** Knowledge scores of microbiome science in different medical sciences student groups

	Adequate (%)	Moderate (%)	Low (%)	P value
Knowledge of microbiome science				
CN	81.2	18.8	0	ns
CLS	83.8	13.5	2.7	
PH	71.0	25.8	3.2	
Knowledge of impaired microbiota and dysbiosis				
CN	40.6	21.9	37.5	ns
CLS	21.6	24.3	54.1	
PH	19.4	32.3	48.3	
Management of the impaired microbiome				
CN	25.0	59.4	15.6	**
CLS	19.0	40.5	40.5	
PH	16.1	19.4	64.5	

Percentage distribution of students with different knowledge levels of microbiome science, impaired microbiota and dysbiosis, and management of impaired microbiome within each medical sciences major (*CN* clinical nutrition, n  = 32; *CLS *clinical laboratory sciences n  = 37; *PH *public health). Chi-square tests were used for statistical analyses (**P  < 0.01, *ns *not significant)

**Table 2:** Correct response rates of microbiome science survey in medical sciences students

	Correct response %
Knowledge of microbiome science	
A. The concept of microbiome	73
1. The term ‘microbiome’ refers to all microorganisms in the human body (yes)	49
2. The term ‘microbiome’ refers only to the bacterial cells living in the human body (no)	42
3. There are microorganisms living naturally in the intestinal tract (yes)	98
4. There are microorganisms living naturally in the respiratory tract (yes)	66
5. There are microorganisms living naturally on the skin (yes)	95
6. There are microorganisms living naturally in the vagina (yes)	88
B. The transfer of microorganisms and links with infection	67
1. All microorganisms can cause an infection (no)	94
2. There are microorganisms in breast milk (yes)	57
3. There are microorganisms present naturally in the food we eat (yes)	83
4. Mothers can transfer microorganisms to infants during breastfeeding (yes)	79
5. Mothers can transfer microorganisms to infants during pregnancy (yes)	67
6. Children exposed to outdoor activities such as farms/gardens get more infections (no)	22
C. The interaction between the intestinal (gut) microbiome and the rest of the body	47
1. There is an interaction between the intestinal and the lung microbiome (yes)	25
2. There is an interaction between the intestinal microbiome and the brain (yes)	38
3. There is an interaction between the intestinal microbiome and the skin (yes)	35
4. The intestinal tract contains the highest number of microbial cells compared to the other systems (yes)	76
5. Microbiome composition is similar for all people (no)	60
Knowledge of impaired microbiota and dysbiosis	
A. The root of dysbiosis	47
1. Dysbiosis refers to altered microbial diversity in the human body (yes)	38
2. Changes in the respiratory microbiome are associated with asthma and allergy (yes)	57
3. Changes in the intestinal microbiome are associated with allergic conditions and inflammatory diseases (yes)	53
4. Changes in the intestinal microbiome are associated with metabolic disorders such as obesity (yes)	40
B. What triggers microbial dysbiosis?	59
1. Microbial dysbiosis can be managed/treated to reduce the risk of allergic disorders (yes)	42
2. Reduced antibiotic use during infancy/perinatally can lower the chances of dysbiosis (yes)	45
3. Consumption of antibiotics can alter the human microbiome (yes)	82
4. Cleaning hands with antimicrobial soap is important to prevent all infections (no)	66
Management of the impaired microbiome	
A. The concept of beneficial bacteria	55
1. Fermented food is a source of beneficial bacteria (yes)	73
2. Dairy products are sources of beneficial bacteria (yes)	85
3. Consumption of “bacterial strain” supplements should be avoided because they might be harmful (no)	30
4. Probiotics are natural antibiotics (no)	22
5. Probiotics are a type of vitamin to improve health (no)	47
6. Probiotics can be used to balance the microbial composition and diversity in the body (yes)	65
7. Probiotics are live bacteria (yes)	63
B. Fecal microbiota transplantation	45
1. Are you familiar with the ‘fecal microbiota transplantation’ procedure (yes)	40
2. Fecal microorganisms can be transplanted or transferred from a healthy to a sick individual (yes)	49

The percentage score of each question represents the frequency of all participants (n  = 100) giving correct responses, while the score of each section represents the average correct response rate of all questions in that section. Correct response (yes/no) to each question was provided in the parentheses

For questions related to the transfer of microorganisms and links with infection, 67% of all students provided correct answers to the survey. While most of the participants correctly agreed that all microorganisms are directly linked to infections (94%) and could be transferred during food consumption (83%), only 22% correctly indicated that exposure to outdoor activities such as farming or playing in gardens does not predispose children to more infections. Most participants correctly agreed that microorganisms could be transferred through breastfeeding (79%); however, fewer participants correctly agreed that microorganisms are present in breast milk (57%) and during pregnancy (67%) (Table 2).

In contrast to the previous two topics, only 47% of all students provided correct responses to questions on interaction between the intestinal (gut) microbiome and the rest of the body. Although 76% of respondents correctly agreed that the intestinal tract contains the highest number of microbes among all human body parts, their knowledge of the potential link or interaction between the intestinal microbiota and the microbiota of other parts of the body was low. In fact, only 25%, 35%, and 38%, respectively, of all participants correctly responded that the gut microbiome could interact with its counterparts in the respiratory tract, skin, and brain. Lastly, a total of 60% of the respondents correctly responded that microbiome compositions differ across individuals (Table 2).

### Knowledge of impaired microbiota and dysbiosis

Unlike microbiome science knowledge, most students in all three medical sciences majors had moderate-to-low overall knowledge of impaired microbiota and dysbiosis. Notably, the CN major had the highest frequency of students with adequate knowledge about this topic. However, no significant differences in the overall knowledge level were found among CN, CLS, and PH students (Table 1).

Regarding questions related to the root cause of dysbiosis, only 47% of students provided overall correct responses. Specifically, 38% of participants were familiar with the term “dysbiosis”. Those who correctly agreed that changes in the microbiome are linked to metabolic syndromes, inflammatory diseases, and allergy/asthma were 40%, 53%, and 57% students, respectively. Regarding factors triggering dysbiosis, a slightly higher percentage of students provided correct responses (59%). Although most of the participants correctly agreed that antibiotics disrupt the microbiome (82%) and that cleaning hands with soap will not prevent all infections (66%), only 42–45% correctly noted that early exposure to antibiotics during infancy might lead to allergic disorders later in life and that dysbiosis management could be employed to treat allergic diseases (Table 2).

### Knowledge of impaired microbiome management

Consistent with the above findings on the knowledge of dysbiosis, most students had a moderate to low level of overall knowledge of the management of this condition. Notably, the knowledge level on the management of the impaired microbiome was significantly higher in the CN group; only 15.6% of students in this major had a low knowledge level compared to those in CLS and PH majors (40.5% and 64.5%, respectively) (Table 1).

Regarding beneficial bacteria, 55% of respondents provided correct responses. CN students had the highest correct response rate (72.8%) compared with CLS and PH students (51% and 41.5%, respectively). While most respondents correctly believed that fermented food and dairy products could potentially be used as sources of beneficial bacteria (73% and 85%), respectively; only 30% of the participants correctly believed that consumption of supplements that contain microorganism cultures are not harmful. Furthermore, 63–65% of respondents correctly noted that probiotics are live bacteria and could be used to balance the microbiome composition and diversity of the body. However, only 47% correctly disagreed that probiotics are vitamins, and only 22% correctly noted that probiotics are not antibiotics (Table 2).

For questions related to FMT, 45% of all respondents provided correct survey answers. CLS students had the highest correct response rate (59.5%) compared with CN and PH students (32.8% and 38.7%, respectively). Specifically, only 40% of respondents were familiar with the concept of FMT, while 49% correctly thought that it is possible to transfer the microbial flora from healthy to sick individuals.

## Discussion

To our knowledge, this is the first study to assess microbiome-related knowledge among medical sciences students in Saudi Arabia. The survey revealed that CLS, CN, and PH students possess adequate knowledge about many general topics of microbiome science. However, they are not sufficiently informed about microbial dynamics in different tissue compartments. These observations are consistent with previous studies, which demonstrated that participants with a medical background are aware of the microbiome concept but lack enough understanding of novel discoveries in this rapidly evolving field. For instance, general knowledge of microbiome science was adequate; however, the knowledge level on tissue-specific microbial composition, such as those in the oral cavity, was low in participants with a medical background in previous studies in the UAE and US [18, 19].

Secondly, the survey results demonstrated that medical sciences students in this study lack sufficient knowledge about microbial dysbiosis in the context of disease pathogenesis and management, particularly regarding probiotics and FMT. In this regard, this finding is consistent with previous studies in the Philippines and Iran, which noted that while the majority (51–88%) of the participants are familiar with the concept of probiotics, few have utilized or were aware of probiotics as treatments for various health conditions [20, 21]. Similarly, the low level of FMT knowledge among the participants in this study was similar to previous findings in Romania and China [22, 23]. This low level of knowledge about therapeutic applications of microbes might be attributed to the insufficient exposure to education on this topic and the absence of widespread application of these procedures for managing various health conditions. In fact, with the rapidly growing information about microbiome science, most textbooks and reference books are not updated, particularly those related to its application to patient care.

Another noteworthy finding from this survey is that CN students appeared to have better knowledge about the management of the impaired microbiome. These findings are consistent with the reported statistics in the survey of students with medical sciences majors in the Philippines, in which CN students scored 42.3% while PH students scored 29% on questions related to probiotics as a treatment for various illnesses [20]. This finding might be attributed to the exposure of CN students to the “Functional Food” course, which detailed the process of food fermentation and the concept of probiotics and their associated health benefits. More importantly, this finding provides important proof that curricular exposure could significantly enhance the knowledge of medical sciences students about the clinical application of microbiome science.

Given the increasing prevalence of asthma and allergy in Saudi Arabia, several questions in this survey were designed to evaluate the participants’ knowledge of the involvement of microbes in these health conditions. While more than 90% of the students are aware of the presence of microbes in other mucosal surfaces, a substantially lower portion of the study participants (57–66%) know that microbes exist in the respiratory tract and that dysbiosis can result in allergic diseases. Similarly, the students’ knowledge about managing the impaired microbiome in the context of allergic disease development and intervention was also inadequate. In fact, most students were not aware that antibiotic misuse during early life may cause allergic disorders later during childhood and continue throughout adulthood and that probiotics could be employed to treat these health conditions. Furthermore, most students believed that outdoor exposure might be harmful to children in the context of infection susceptibility. This misbelief is particularly important in implementing prophylactic measures against the development of allergic diseases, as frequent exposure to environmental stimuli has been suggested to reduce the risk of developing these disorders [30–32]. Therefore, educational materials on the role of microbes in the development of allergic diseases and the clinical implications are critical to improving the knowledge of medical sciences students whose future careers will be involved in providing direct care and formulating healthcare policies for emerging public health concerns in Saudi Arabia.

In medical education, needs’ assessments represent the basis of curriculum development and updating the syllabus [33]. Therefore, identifying the required competency of medical sciences graduates is a fundamental step in developing competency-based curricula [34]. In the light of these findings, a frequent update of the medical sciences curriculum might be necessary to provide the students with cutting-edge knowledge in microbiome science. Specifically, observations about different shortcomings in microbiome science knowledge could be translated into recommendations to update the content of the microbiology courses, including those related to the tissue-specific microbial repertoires, the role of microbial dysbiosis in disease development, and the therapeutic manipulation of the microbiome. Additionally, given the lack of academic reference books on this topic, the learning outcomes of the intended course should be expanded to accommodate more seminar sessions to discuss the latest scientific and technological discoveries in the microbiome, as well as the role of the microbiome in non-infectious illnesses.

The current study has several limitations. First, the study cohort, in which the participants are recruited from three majors in a single academic institution, is relatively small. Therefore, it remains unknown whether a similar level of microbiome science knowledge exists among students with similar majors across Saudi Arabia. Secondly, since all of the participants in this study were females, the knowledge of microbiome science in male medical science students remains unknown. This sampling bias was a result of the absence of male students, as they are not enrolled in the CN, CLS, and PH majors at our university. Thirdly, while the study revealed informative statistics about the shortcomings in microbiome knowledge among different medical sciences majors, the comparisons were carried out among students with varying exposure to this topic through their curriculum. For instance, as mentioned above, the estimated microbiome-related content in the curriculum for PH and CN is 5–10%, while that for CLS students is 15–20%. Lastly, the study did not address whether the observed level of microbiome knowledge of the students meets the learning outcomes of each program, as well as the students’ future professions. Therefore, future studies should include a larger sample size, with both male and female students, and compare each program across different universities in Saudi Arabia. The knowledge evaluation of such studies should also consider the specific program learning outcomes and the job market requirements. Additionally, the microbiome knowledge of students with medical and pharmacy majors can also be similarly assessed. This will allow these future clinicians and pharmacists to implement this knowledge in their daily practice and discuss with their patients the root causes of disease and the available options to restore microbial balance in relevant illnesses.

## Conclusions

The collective results of the current study not only revealed existing gaps in microbiome knowledge among medical sciences students but also highlighted the need to incorporate emerging human microbiome research findings into the medical program curricula. Furthermore, these findings suggest that additional learning outcomes to evaluate the students’ competency concerning this topic should be implemented. Since improving the quality of life, managing health, and preventing diseases are core learning competencies of all health programs, the microbiome can be viewed as a tool to improve lives, manage symptoms, and restore the physiological functions of patients suffering illnesses of emerging public health concerns, such as allergies and asthma.

## Supplementary Information

**Additional file 1: **QuestionPro-Survey microbiome knowledge study.

## Data Availability

Raw data are available upon reasonable request.

## Abbreviations

CLS: Clinical laboratory sciences
CN: Clinical nutrition
FMT: Fecal microbiota transplantation
PH: Public health
UAE: United Arab Emirates
US: United States
