# Supplementary material for: Evaluating the knowledge on microbiome and dysbiosis in allergic diseases among medical sciences students in Saudi Arabia
Source: Clin Mol Allergy. 2022 Jan 31;20:2. doi: 10.1186/s12948-022-00168-x (PMC8802525; doi:10.1186/s12948-022-00168-x)
Supplement: Supplementary file 1 — Additional file 1: QuestionPro-Survey microbiome knowledge study. [file 12948_2022_168_MOESM1_ESM.pdf]

Microbiome Science

Hello: You are invited to participate in this survey. It will take approximately [10] minutes to complete the questionnaire. Your participation in this study is completely voluntary. There are no foreseeable risks associated with this project. However, It is very important for us to learn your opinions. Your survey responses will be strictly confidential and data from this research will be reported only in the aggregate. Your information will be coded and will remain confidential.
Thank you very much for your time and support. If you wish to continue, please start with the survey now by clicking on the NEXT button below.

Section 1: Demographic and clinical characteristics of the participants

Age (Years):

Gender
1. Male
2. Female

What is your Specialty?
1. CT
2. CN
3. CLS
4. RC
5. Public Health
6. Clinical Pharmacy
7. Other (…………….)

What is your Educational status?
1. Student
2. Intern

Section 2: Knowledge on the Human Microbiome

The term 'Microbiome' refers to all microorganisms in the human body?
1. Yes
2. No
3. Don't Know

The term 'Microbiome' refers only to the bacterial cells living in the human body?
1. Yes
2. No
3. Don't Know

Microbiome Science

All microorganisms can cause an infection?

1. Yes
2. No
3. Don't Know

There are microorganisms living naturally in the intestinal tract ?

1. Yes
2. No
3. Don't Know

There are microorganisms living naturally in the respiratory tract ?

1. Yes
2. No
3. Don't Know

There are microorganisms living naturally on the skin ?

1. Yes
2. No
3. Don't Know

There are microorganisms living naturally in the vagina

1. Yes
2. No
3. Don't Know

There are microorganisms in breast milk ?

1. Yes
2. No
3. Don't Know

There are microorganism present naturally in the food we eat ?

1. Yes
2. No
3. Don't Know

Mothers can transfer microorganisms to infants during breast feeding ?

1. Yes
2. No
3. Don't Know

Microbiome Science

Mothers can transfer microorganisms to infants during pregnancy ?

1. Yes
2. No
3. Don’t Know

Children exposed to outdoor activities such as farms/gardens get more infections ?

1. Yes
2. No
3. Don't know

There is an interaction between the intestinal and the lung microbiome ?

1. Yes
2. No
3. Don't know

There is an interaction between the intestinal microbiome and the brain ?

1. Yes
2. No
3. Don’t Know

There is an interaction between the intestinal microbiome and the skin ?

1. Yes
2. No
3. Don’t Know

The intestinal tract contains the highest number of microbial cells compared to the other human body systems ?

1. Yes
2. No
3. Don’t Know

Microbiome composition is similar for all people ?

1. Yes
2. No
3. Don’t Know

Section 3: Knowledge on Dysbiosis

Dysbiosis refers to the altered microbial diversity in the human body?
1. Yes
2. No
3. Don't know

Changes in respiratory microbiome are associated with asthma and allergy ?
1. Yes
2. No
3. Don’t Know

Changes in intestinal microbiome are associated with allergic conditions and inflammatory diseases?
1. Yes
2. No
3. Don’t Know

Changes in intestinal microbiome are associated with metabolic disorders such as obesity?
1. Yes
2. No
3. Don’t Know

Microbial dysbiosis can be managed/treated to reduce the risk of allergic disorders ?
1. Yes
2. No
3. Don't know

Reduced antibiotic use during infancy/perinatal can lower the chances of dysbiosis?
1. Yes
2. No
3. Don’t Know

Consumption of antibiotics can alter the human microbiome ?
1. Yes
2. No
3. Don’t Know

Cleaning hands with antimicrobial soap is important to prevent all infections?
1. Yes
2. No
3. Don’t Know

Section 4: Perception of Probiotics

Fermented food is a source of beneficial bacteria ?
1. Yes
2. No
3. Don’t Know

Dairy products are sources of beneficial bacteria ?
1. Yes
2. No
3. Don’t Know

Consumption of nutritional supplement labeled as ‘’bacterial strains’’ should be avoided because they might be harmful ?
1. Yes
2. No
3. Don’t Know

Probiotics are natural antibiotics ?
1. Yes
2. No
3. Don’t Know

Probiotics are a type of vitamins to improve health?
1. Yes
2. No
3. Don’t Know

Probiotics can be used to balance the microbial composition and diversity in the body?
1. Yes
2. No
3. Don’t Know

Probiotics are live bacteria ?
1. Yes
2. No
3. Don’t Know

Are you familiar with the ‘fecal microbiota transplantation’ procedure?
1. Yes
2. No
3. Don’t Know

Microbiome Science

Fecal microorganisms can be transplanted (transferred) from a healthy to sick individual ?

1. Yes
2. No
3. Don’t Know
